# Visual change as the presenting symptom of a suspected metastatic brain lesion in esophageal cancer: A case report

**DOI:** 10.1097/MD.0000000000036014

**Published:** 2023-11-24

**Authors:** Yun-Chen Hsieh, Jian-Sheng Wu

**Affiliations:** a Department of Ophthalmology, Changhua Christian Hospital, Changhua, Taiwan; b Department of Post-Baccalaureate Medicine, College of Medicine, National Chung Hsing University, Taichung, Taiwan.

**Keywords:** brain neoplasms, esophageal neoplasms, vision disorders

## Abstract

**Introduction:**

Esophageal cancer is the seventh most common malignancy worldwide and the sixth leading cause of cancer mortality with an overall survival rate of <20%. Esophageal cancer frequently metastasizes to distant lymph nodes, lungs, liver, and bones. Cerebral metastases originating from esophageal cancer are rare and often carry a poor prognosis as do most all metastatic lesions in esophageal cancer.

**Case presentation:**

In this report, we describe a 55-year-old patient with past history of esophageal carcinoma who presented with blurred vision after taking ethambutol for tuberculosis. Ethambutol-induced optic neuropathy was the lead diagnosis. Initial vision testing was normal so additional testing with visual field examination was warranted. The visual field examination revealed homonymous hemianopsia. Subsequent magnetic resonance imaging of his brain, demonstrated a focal lesion, consistent with but not diagnostic of a brain metastasis likely from his primary esophageal malignancy.

**Conclusion:**

We conclude that a careful review of the medical history and comprehensive assessment are essential in establishing an obscure clinical diagnosis especially in the event that an uncommon metastatic lesion is encountered.

## 1. Introduction

Gastrointestinal malignancies constitute 26% malignancies globally and 35% malignancy related mortality, with approximately 4.8 million new cases and 3.4 million deaths each year.^[1]^ Although gastrointestinal cancers are frequently diagnosed, the occurrence of brain metastases (BMs) is infrequent. Prior studies show that the majority of BMs originating from gastrointestinal malignancies are related to colorectal cancers (1%–4%). Detection of BMs in esophageal carcinoma is (1.4%–1.8%), in gastric carcinomas (0.16%–0.69%), in hepatic carcinomas (1.3%–2.9%), in pancreatic carcinoma (0.1%–0.3%), and in carcinoma of the gallbladder (<0.5%) which have all been previously established.^[2]^ Brain metastases cause a wide range of symptoms based on the location, size, and rate of growth of the tumors, with the most common symptoms including headaches, mental changes, or focal neurologic deficits including changes in vision.

This report presents an esophageal cancer patient who presented with homonymous hemianopsia while taking ethambutol, and was ultimately found to have suspected metastatic esophageal cancer in his brain and later, in multiple organs.

## 2. Case presentation

A 55-year-old man presented with blurred vision after being treated for pulmonary tuberculosis with ethambutol for 2 months. He had a prior history of esophageal cancer 2 years prior at another hospital, which was treated with radiotherapy and chemotherapy. Pulmonary tuberculosis was diagnosed 2 months ago and he was treated with isoniazid, pyrazinamide, and rifampin combination tablet (Rifater) and ethambutol (Epbutol, 1200 mg/day) for 2 months.

**Figure 1.**
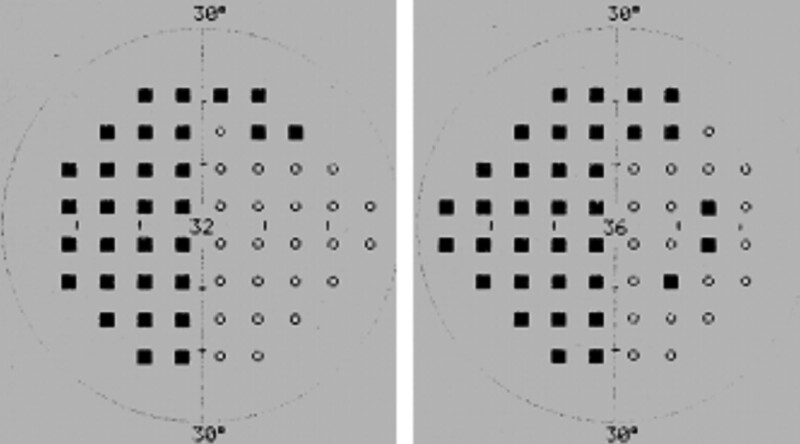
Oculus automated perimetry demonstrates a left homonymous hemianopsia.

**Figure 2.**
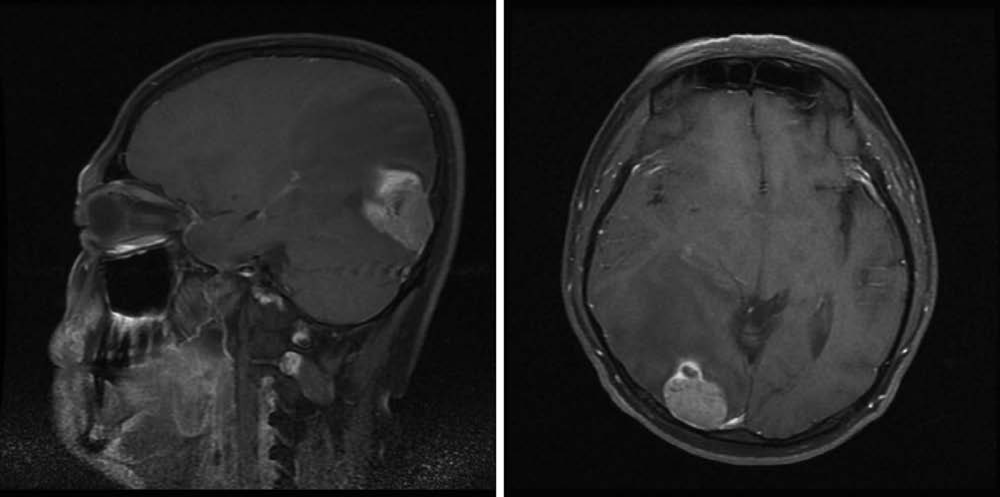
Brain magnetic resonance imaging demonstrates a focal lesion at the right occipital lobe with compression of the posterior horn and of the right lateral ventricle.

**Figure 3.**
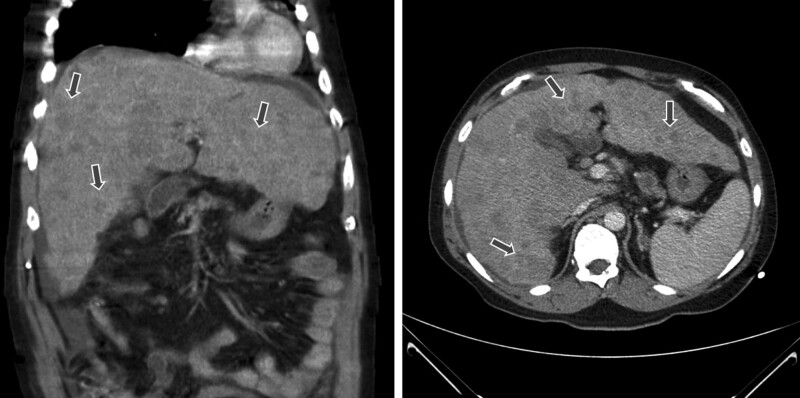
Chest computed tomography scan contains multiple foci (arrows) in both lobes of the liver.

His physical exam revealed that corrected visual acuity was 20/20 oculus dexter and 20/25 oculus sinister. Color vision, pupil response, and fundoscopic examination were all normal. The patient affirmed that the visual changes onset began after ingesting the prescribed ethambutol. Early toxicity was suspected and visual field examination was arranged. A left homonymous hemianopia was revealed by Oculus automated perimetry (Fig. 1). Brain magnetic resonance imaging (MRI) demonstrated a focal lesion in the right occipital lobe with compression of the posterior horn and of the right lateral ventricle (Fig. 2). Review of his past medical history of esophageal carcinoma, led to the suspicion of a brain metastasis from his prior esophageal carcinoma. Additional diagnostic studies were recommended, which he declined and was ultimately lost to follow-up. Three months later, he presented to the emergency room due to an acute onset of dyspnea. Massive pleural effusion with left lung collapse was found. The pleural effusion was tapped but no malignant cells and no pathogens were detected. Chest computed tomography scan showed multiple low-density lesions in both lobes of the liver (Fig. 3) suspicious for metastatic disease. Bronchoscopy revealed that the left main bronchus was completely occluded by tumor and biopsy was performed. Pathologic evaluation revealed no evidence of malignancy. Unfortunately, he died from a sudden onset of bradycardia and hypotension on the seventh day of hospitalization.

## 3. Discussion

Esophageal cancer is the seventh most common type of cancer globally and accounts for the sixth leading cause of cancer mortalities accounting for over 500,000 deaths annually. The incidence of esophageal cancer varies widely with geographic regions and some areas have an incidence as high as 160 to 540 people per 100,000.^[3]^ The organs mostly likely to be affected by metastatic spread of esophageal cancer are lymph nodes, liver, peritoneum, lung, and adrenal glands. Brain metastasis originating from esophageal cancer have been regarded as rare, with reported incidences ranging from 0% to 6% in recent studies.^[4]^ Recent studies have also discovered that esophageal metastatic cancer cells can leave the primary esophageal cancer site via 3 pathways: lymphatic, venal, or arterial which may lead to distal unexpected metastases in some patients.^[5]^

Due to the rare incidence of BMs in esophageal cancer, neither routine intracranial screening nor firm guidelines for treatment exist. Currently, therapeutic strategies for BMs include surgical resection, whole brain radiotherapy, and stereotactic radiosurgery.^[4]^ Regardless of the mode of treatment, the survival of patients with BMs from gastrointestinal malignancies is inferior than those with BMs from other types of cancer. Furthermore, BMs developed from esophageal cancer are rare and arise in the advanced stages of the disease. Prognosis is often dismal with overall survival after BMs of a mere 5 months.^[2]^

Tuberculosis is considered to be a commonly transmittable diseases worldwide including in Taiwan. Though the incidence of new cases decreases annually in Taiwan, there were still over 7000 tuberculosis cases in 2021.^[6]^ Ethambutol is a bacteriostatic antibiotic used in the treatment of tuberculosis and optic neuropathy is one of its most notable and disastrous complications. It occurs in 1% of patients taking ethambutol at the World Health Organization recommended doses. Most patients complain of visual symptoms within the first 9 months of treatment and they typically experience bilateral, subacute, painless, and symmetric loss of central vision, which may sometimes be described as having cloudy vision and having difficulty both in reading and in distinguishing color. Assessments such as visual acuity, visual field, color vision testing, and dilated fundoscopic examination should be included in the diagnostic evaluation.^[7]^

In this case, the patient underwent a series of testing. The initial visual acuity test was negative, which made ethambutol-induced optic neuropathy less likely. Visual field testing was subsequently completed and detected homonymous hemianopsia, which is frequently associated with brain neoplasms, as in this case. The patient received no treatment for the brain lesion and presented 3 months after this diagnosis. The patient’s condition deteriorated rapidly, shortly after he presented, and he expired due to widespread multiorgan metastases, which led to multisystem-organ-failure. This case not only highlights the rarity of BMs in esophageal cancer, but also demonstrates the rapidity of disease progression in esophageal cancer. A major limitation of this study is the lack of histological diagnoses of any of the distant metastases. We can only speculate that the cerebral metastasis is most likely to be esophageal in origin and far less likely to have occurred from multiple malignancies such as lung cancer and liver cancer; although that possibility cannot be entirely excluded.

This case began with a man under treatment for tuberculosis and with a past history of esophageal carcinoma who began having disturbances in his vision. He was determined to not have ethambutol-induced optic neuropathy, but instead had homonymous hemianopsia which is common in mass lesions in the brain. This led to the performance of a brain MRI. His brain MRI confirmed the presence of a mass lesion. While BMs from esophageal cancer are rare, physicians should be aware of the possibility in patients with a history of esophageal or other gastrointestinal tract related cancers. Certainly, a complete medical history and thorough examination are essential to obtaining a precise clinical diagnosis and unmasking any obscure problems.

## Author contributions

**Conceptualization:** Yun Chen Hsieh, Jian-Sheng Wu.

**Supervision:** Jian-Sheng Wu.

**Writing – original draft:** Yun Chen Hsieh.

**Writing – review & editing:** Jian-Sheng Wu.
